# Influencing of particle size, calcination, and mineral enrichment of local calcites on productivity and economic efficiency of quails (*Coturnix japonica*)

**DOI:** 10.5455/javar.2026.m1063

**Published:** 2026-06-22

**Authors:** Khalil Khalil, Dwi Ananta, Andri Andri, Ridho Kurniawan Rusli

**Affiliations:** 1 Department of Animal Nutrition and Feed Technology, Faculty of Animal Science, Universitas Andalas, Campus II Payakumbuh, Jl. Rasuna Said Kubu Gadang, Payakumbuh 26211, West Sumatra, Indonesia; 2 Department of Animal Production and Technology, State Agricultural Polytechnic of Payakumbuh, Jl. Raya Tanjung Pati Km. 7, Limapuluh Kota 26271, West Sumatra, Indonesia; 3 Department of Livestock Business and Development, Faculty of Animal Science, Universitas Andalas, Limau Manis, Padang 25163, West Sumatra, Indonesia; 4 Department of Animal Nutrition and Feed Technology, Faculty of Animal Science, Universitas Andalas, Limau Manis, Padang 25163, West Sumatra, Indonesia

**Keywords:** calcite grit, calcination, *Coturnix japonica*, net return, physical property

## Abstract

**Background:** Small-scale quail farmers commonly use semi-self-mixed diets that may reduce mineral availability and compromise laying performance. Locally sourced calcite could provide a cheaper, more effective alternative to commercial mineral premixes.

**Objectives:** This study evaluated the effects of locally formulated calcite-based mineral supplements that differed in particle size, calcination, and mineral enrichment on the productivity, physiological responses, and economic efficiency of laying quails.

**Materials and Methods:** Five local calcite-based mineral formulas were produced from rock flour and oyster shell meal in raw or calcined forms, with or without mineral enrichment. A total of 432 laying quails were assigned to six dietary treatments, including a commercial mineral premix and five local mineral supplements incorporated at 5% of the basal diet for eight weeks. Productive performance, eggshell quality, blood mineral profile, physical characteristics of the minerals, and economic returns were evaluated.

**Results:** Local calcite-based minerals significantly improved egg production, egg mass, feed conversion ratio, and net return compared with the commercial premix (*p* < 0.05). Calcite grit (Cag) produced the highest productivity and profitability. Its coarse particle size and greater density likely enhanced calcium retention and sustained mineral release for eggshell formation. Although calcination and mineral enrichment increased serum phosphorus levels, they did not further improve production performance or economic efficiency.

**Conclusions:** Calcite grit is an effective and economically superior mineral supplement for laying quails. Physical characteristics, particularly coarse particle size and density, contributed more to productive performance than calcination or mineral enrichment.

## 1. Introduction

Quail eggs are an important source of animal protein in Indonesia after chicken and duck eggs. Domestic quails (*Coturnix japonica*) exhibit high reproductive efficiency, reaching sexual maturity at 6–10 weeks of age and producing approximately 200–300 eggs annually [1]. Because of their small body size and limited feed intake capacity, laying quails require diets with high nutrient density and balanced mineral supplementation to sustain egg production and maintain eggshell quality [2].

Layer quail production in Indonesia is dominated by smallholder farms with flock sizes of approximately 3,000–6,000 birds [3]. Commercial complete feeds are commonly used; however, to reduce feeding costs, farmers often dilute these diets with inexpensive local ingredients, such as ground yellow corn and rice bran (semi-self-mixing). This practice may reduce nutrient density, particularly mineral availability, because these ingredients have low mineral concentrations, while rice bran’s high phytate content further limits mineral absorption [4]. Often, commercial mineral premixes are added without prior evaluation of the nutrient composition of semi-self-mixed diets, leading to imprecise mineral supplementation, potential nutrient deficiencies, and increased production costs [5].

Calcium (Ca) is the most important macromineral for laying quails because it plays a critical role in skeletal maintenance and eggshell formation. During the laying period, quails require 3–4% dietary Ca, substantially higher than their phosphorus requirement of 0.5–0.8% [6]. In addition to calcium concentration, particle size and physical characteristics influence mineral utilization. Coarse calcium particles are beneficial because they remain longer in the gizzard and provide sustained calcium release during nocturnal eggshell formation [7]. Therefore, mineral supplements with appropriate physical properties and high bioavailability are essential for optimal laying performance.

Indonesia possesses abundant natural calcite resources, including limestone deposits and oyster shells, which have potential as affordable local mineral sources for poultry feed. Previous studies demonstrated that rock flour and oyster shell meal can serve as effective calcium sources for poultry diets [8, 9]. Their nutritional value may be further improved through calcination to enhance mineral bioavailability and to enrich them with essential minerals such as phosphorus (P), zinc (Zn), and copper (Cu), thereby producing a more balanced mineral supplement [9, 10]. The use of locally formulated mineral supplements might reduce feed costs and improve profitability in poultry production systems.

The present research aimed to formulate local calcite-based mineral supplements derived from raw or calcined rock flour and oyster shell meal and to evaluate the effects of calcination and mineral enrichment on production performance, physiological responses, and economic efficiency in laying quails. We hypothesized that locally formulated calcite-based minerals would perform comparably to or better than commercial mineral premixes while providing greater economic benefits for small-scale quail production.

## 2. Materials and Methods

### 2.1. Ethical approval

The present research was reviewed and approved by the Animal Care Ethics Committee of Faculty of Medicine of Universitas Andalas under protocol number 437/UN16.2/KEP-FK/2025.

### 2.2. Design and preparation of locally derived calcium mineral formulas

Five calcite-based mineral formulas were prepared from local rock flour and oyster shell meal in either raw or calcined form, and some formulas were further enriched with selected macro- and microminerals. These were (1) calcite flour (Caf): 100% raw rock flour; (2) calcite grit (Cag): a mixture of 70% coarse-ground raw rock and 30% oyster shell grit; (3) calcite phosphate (CaP): raw rock flour and oyster shell meal combined with steamed bone meal; (4) enriched calcite (eCa): raw rock flour mixed with oyster shell meal and enriched with supplemental macro- and trace minerals; and (5) enriched calcined calcite (ecCa): calcined rock and calcined oyster shell flour further enriched with supplemental macro- and trace minerals. The enriched formulas (eCa and ecCa) were prepared by incorporating dicalcium phosphate (DCP), iodized salt, zinc sulfate heptahydrate (ZnSO_4_·7H_2_O), and copper sulfate pentahydrate (CuSO_4_·5H_2_O) according to the nutrient requirements of laying quails [6]. DCP was included to improve calcium and phosphorus balance for eggshell mineralization [11]. In contrast, Zn and Cu were added because of their important roles in enzyme activity, mineral metabolism, eggshell formation, and reproductive performance in laying birds [12]. The composition and specifications of each formula are listed in Table 1.

**Table 1.** Design of locally derived calcium mineral formulas.

No	Formula	Abbreviation	Composition of raw materials	Specification
1	Calcite flour	Caf	100% raw rock flour meal	Ca source, fine particle size
2	Calcite grit	Cag	70% coarse-ground raw rock flour and 30% oyster shell (30%) mix	Ca source, coarse grit
3	Calcite phosphate	CaP	70% of raw rock flour and 15% of oyster shell meal mixed with 15% of steamed bone meal	Ca and P source
4	Enriched calcite	eCa	34% of raw rock flour, 10% of oyster meal, 15% of rock and oyster shell grit, 20% of bone meal, 15% of DCP, 5% kitchen salt, and enriched with 1% trace minerals	Complete mineral supplement
5	Enriched calcined calcite	ecCa	34% of calcined rock and 10% calcined oyster shell, 15% rock and oyster shell grits, 20% bone meal, 15% of DCP, 5% kitchen salt, enriched with 1% trace minerals*	Complete supplement, calcined

Raw rock flour and coarse grit were sourced from local rock mining sites in West Sumatra, Indonesia. Oyster shell meal and grit were produced from underutilized blood clam shells (*Anadara antiquata* L.) collected from coastal areas following the procedures described by Khalil et al. [8]. The calcined rock and calcined oyster shell flour were prepared following the procedure of Khalil et al. [9]. Small rock boulders and intact oyster shells were calcined in a modified metal drum furnace at 800–900 °C for 48 h until the materials became completely whitened and brittle, indicating thermal decomposition of carbonate compounds. The calcined materials were subsequently ground and sieved (<150 µm) to produce fine calcined flour.

Calcination eliminates water and organic impurities and converts calcium carbonate into calcium dioxide (CaO) [13]. Calcination increased essential mineral concentration, the percentage of finer particles, and surface area [9, 13, 14], resulting in better digestibility and absorption of the essential mineral.

Steamed bone meal was prepared using inedible cattle bones derived from a local slaughterhouse, as described by Khalil et al. [15]. Fresh slaughterhouse bones were washed, pressure-boiled for 4 h, and rinsed to remove fat and soft tissue. The cleaned bones were sun-dried, broken into 2–3 cm chips, soaked in 10% lime water for 5 days, oven-dried at 60 °C for 48 h, and finally coarse-ground to produce bone meal.

### 2.3. Analysis of minerals, particle size, and physical properties

Six representative samples of each mineral type were collected to determine the concentrations of macro-minerals (Ca, P, Mg, K, and Na) and trace elements (Fe, Zn, Cu, and Mn). Sample preparation and digestion were performed according to the AOAC standard procedures [16], and minerals were analyzed using atomic absorption spectrophotometry. The particle size distribution was determined using sieve analysis [9]. The percentage of each particle size fraction was classified into three groups: coarse (≥ 1 mm), medium (0.3–1 mm), and fine (< 0.3 mm) to justify the optimal particle size for laying quails [17]. The particle size distribution was determined via sieve analysis utilizing a Retsch VS 1000 sieve shaker (Retsch GmbH, Germany).

The physical properties related to intake capacity, mixture homogeneity, and stability were measured according to the procedures outlined by Khalil et al. [9]. The bulk density, the tapped bulk density, the specific density, and the angle of repose were measured. Bulk and compacted densities were determined by weighing sample volumes in a 100-mm graduated cylinder before and after tapping. Bulk and compacted densities were calculated by dividing the sample’s mass by its volume before and after tapping. Specific density (gm/ml) was measured using a helium pycnometer. The angle of repose (°) was measured by letting the sample flow from a cylinder onto a flat surface to form a cone-shaped pile. We measured the pile’s height (h) and base diameter (d) and calculated the angle using the formula:


1 \[
\theta = \arctan \left({\frac{{2{\mathrm{h}}}}{d}} \right)
\]

### 2.4. Design of experimental diets and feeding trial

The nutritive value of the designed mineral formulas was evaluated in a 56-day feeding trial. A completely randomized design was used with six experimental diets as treatments and six replicates. Six experimental diets were prepared by supplementing a basal diet with 5% of one of the following: T0: basal diet + 5% commercially mineral premix (CMP) (control); T1: basal diet + 5% calcite flour (Caf); T2: basal diet + 5% calcite grit (Cag); T3: basal diet + 5% calcite phosphate (CaP); T4: basal diet + 5% enriched calcite (eCa); and T5: basal diet + 5% enriched calcined calcite (ecCa).

The basal diets were prepared using the following ingredients: yellow corn, rice bran, fish meal, soybean meal, coconut meal, palm kernel meal, coconut oil, steamed bone meal, kitchen salt, and premix. The nutrient and energy compositions calculated based on the chemical analysis of feed components met the standard requirements of laying quail during the early production period, as recommended by Stanquevis et al. [6]. The experimental diets were formulated to be isonitrogenous and isocaloric, differing only in their mineral sources.

A commercially available mineral premix commonly used by farmers for poultry served as control. The mineral composition, as stated in the label, was as follows: CaCO_3_ (50%), phosphorus (25%), manganese (0.35%), iodine (0.20%), potassium (0.1%), copper (0.15%), sodium chloride (23.05%), iron (0.8%), zinc (0.2%), and magnesium (0.15%). Due to lower Ca and higher P, the control diet had lower Ca and higher phosphorus compared to T1-T5. The control diet (T0) was intentionally formulated based on the feeding practices commonly used by local quail farmers, including the mineral composition and mineral sources typically applied under field conditions. The calculated nutrient and energy content of the experimental diets is presented in Table 2.

**Table 2.** The feed ingredients, nutrients, and energy composition of experimental diets.

Ingredient/nutrient	Experimental diets supplemented with mineral formulas					
	CMP	Caf	Cag	CaP	eCa	ecCa
	T0	T1	T2	T3	T4	T5
Feed ingredients (%)						
- Yellow corn	34.0	36.0	35.0	35.0	35.0	35.0
- Rice bran	5.0	5.0	5.0	5.0	5.0	5.0
- Fish meal	15.0	15.0	15.0	15.0	15.0	15.00
- Soybean meal	20.0	20.0	20.0	20.0	20.0	20.0
- Coconut meal	6.0	6.0	6.0	6.0	6.0	6.0
- Palm kernel meal	5.0	6.0	5.0	5.0	5.0	5.0
- Coconut oil	5.0	4.0	5.0	5.0	5.0	5.0
- Steamed bone meal	4.0	2.0	3.0	3.0	3.0	3.0
- Kitchen salt	0.5	0.5	0.5	0.5	0.5	0.5
- Premix (Top Mix)	0.5	0.5	0.5	0.5	0.5	0.5
- CMP	5.0					
- Caf		5.0				
- Cag			5.0			
- CaP				5.0		
- eCa					5.0	
- ecCa						5.0
Total	100.0	100.0	100.0	100.0	100.0	100.0
Calculated crude nutrients (%) and energy (kcal/kg)						
- Crude protein	20.4	20.8	20.5	20.5	20.5	20.5
- Crude fat	6.3	6.3	7.2	7.2	7.2	7.2
- Crude fiber	4.0	5.1	5.0	5.0	5.0	5.0
- Calcium	2.0	3.0	3.0	3.0	2.9	2.7
- Phosphorus, total	0.8	0.5	0.6	0.6	0.6	0.7
- Ca/P ratio	1.9	4.6	3.9	3.9	3.8	3.4
- Metabolizable energy	2629.1	2629.6	2662.1	2662.1	2662.1	2662.1

Premix supplied per kilogram of diet: Vitamin A: 10,000 IU; Cholecalciferol: 2,000 IU; Vitamin E: 10 mg; Vitamin K₃: 2 mg; Thiamine: 1 mg; Riboflavin: 5 mg; Pyridoxine: 2 mg; Vitamin B12: 0.0154 mg; Niacin: 125 mg; Calcium pantothenate: 10 mg; Folic acid: 0.25 mg; Biotin: 0.02 mg; BHT: 30 mg; Selenium: 0.1 mg; Iron: 40 mg; Copper: 12 mg; Zinc: 120 mg; Manganese: 100 mg; Iodine: 2.5 mg; Cobalt: 0.75 mg.CMP: commercial mineral premix; Caf: calcite flour (Caf); Cag: calcite grit; CaP: calcite phosphate; eCa: enriched calcite; ecCa: enriched calcined calcite (ecCa).

### 2.5. Experimental birds, housing, management, and data collection

A total of 432 eight-week-old laying quails (initial average body weight 99.2 ± 8.9 gm) were randomly allotted into 36 experimental units (cages). Each experimental unit comprised 12 quails housed in cages (75 × 45 × 50 cm), providing a stocking density of approximately 281 cm²/bird. The cages were maintained in a well-ventilated wire-mesh room, with adjustable covers used at night when required. Each dietary treatment was replicated six times using a completely randomized design (CRD). The trial commenced when the flock reached approximately 20% egg production. The birds were housed in battery cages and provided ad libitum access to feed and water.

Throughout the experiment, the following bird performance parameters were recorded or calculated: feed intake, egg mass, feed conversion ratio (FCR), egg weight, egg index, and eggshell quality (weight, percentage, and thickness). At the end of the experimental period, one bird with body weight closest to the replicate mean was selected from each replicate (six birds per treatment) and fasted for approximately 12 h before sampling. The selected quails were slaughtered by placing them in a blood cone to collect blood. The birds were slaughtered in accordance with approved halal slaughter procedures commonly used in poultry processing. Birds were gently restrained in a blood cone, and a rapid, deep neck incision was immediately performed using a sharp knife to sever the major blood vessels, trachea, and esophagus simultaneously, thereby minimizing handling time and distress.

Approximately 3–4 ml of blood was immediately collected using 5-ml disposable syringes and transferred into heparinized tubes for plasma separation. Samples were centrifuged at 3,000 rpm for 15 min (LMC-3000, Grant Instrument, UK), and the plasma was collected into sterile vials for analysis of calcium (Ca), phosphorus (P), magnesium (Mg), and total protein [18] using an auto-analyzer (Mindray) with commercial assay kits. The liver and gizzard weights were recorded.

### 2.6. Economic analysis

A partial budget analysis was performed to assess the economic feasibility of the different mineral supplements. Partial budgeting is a tool used to evaluate the implications of a proposed change in a business operation by comparing the benefits and costs of implementing an alternative to those of the current practice [19, 20].

The analysis included the following steps [20]:

- Total Gross Return (IDR/bird): Calculated as the sum of income from egg sales, quail carcasses, and manure.
- Total Variable Cost (IDR/bird): Calculated as the sum of feed and capital costs.
- Net Return (IDR/bird): Derived by subtracting total variable cost from total gross return.
- Marginal Analysis: Assessed marginal cost, marginal net return, and marginal rate of return to evaluate the economic efficiency of switching between treatments.
- Dominance Analysis: Feeding treatments were classified as dominated if they generated lower or similar net returns but required higher variable costs compared with other dietary treatments, indicating lower economic efficiency.

### 2.7. Statistical analysis

All data were tested for normality (Shapiro-Wilk test) and homogeneity of variances (Levene’s test) before being analyzed using a one-way analysis of variance. Significant differences among means were separated using Duncan’s Multiple Range Test (DMRT). Statistical significance was set at *p* < 0.05. All statistical analyses were performed using the Statistical Package for the Social Sciences (SPSS) version 18.0.

## 3. Results

### 3.1. Mineral composition of calcite-based formulas

Table 3 shows the average mineral concentrations of the five calcite-based formulations. Among the macro minerals, the Ca content was the highest in the Caf, whereas the ecCa exhibited a significantly lower concentration. The Ca concentration was highest in Caf because it consisted predominantly of calcite material. In contrast, ecCa showed a lower Ca concentration due to partial replacement of calcite with supplemental minerals during the enrichment process. An inverse relationship was observed for P, with the ecCa formulation being most abundant, followed by eCa and CaP. The non-enriched groups (Caf and Cag) contained the lowest P. In terms of other macro minerals, K was highest in Caf, CaP, and eCa, whereas the Na and S levels were highest in the ecCa group. Mg was most prevalent in Caf and was significantly lower in Cag.

**Table 3.** Average mineral concentrations (mean ± SD) for calcite-based formulations.

Mineral	Caf	Cag	CaP	eCa	ecCa	SEM	*p*-value
Macro minerals (gm/kg)							
- Ca	399.37^a^ ± 3.58	372.30^b^ ± 7.56	369.00^b^ ± 3.06	362.20^c^ ± 5.73	327.90^d^ ± 4.79	2.122	0.000
- P	0.05^d^ ± 0.00	0.08^d^ ± 0.01	10.61^c^ ± 1.24	12.72^b^ ± 0.24	20.93^a^ ± 1.87	0.411	0.000
- K	0.28^ab^ ± 0.00	0.05^d^ ± 0.01	0.30^a^ ± 0.03	0.28^b^ ± 0.00	0.11^c^ ± 0.01	0.006	0.000
- Na	0.09^b^ ± 0.00	0.46^b^ ± 0.01	1.14^b^ ± 0.02	3.58^b^ ± 1.13	12.65^a^ ± 6.75	1.249	0.001
- Mg	8.27^a^ ± 0.06	2.20^d^ ± 0.01	6.77^b^ ± 0.19	6.97^b^ ± 0.15	4.43^c^ ± 0.26	0.066	0.000
- S	0.93^c^ ± 0.12	0.19^e^ ± 0.09	0.71^d^ ± 0.05	1.24^b^ ± 0.20	2.56^a^ ± 0.08	0.049	0.000
Microminerals (ppm)							
- Fe	1607.74^b^ ± 105.37	1798.02^a^ ± 22.68	1830.12^a^ ± 112.74	1734.37^a^ ± 74.41	817.31^c^ ± 50.03	33.169	0.000
- Cu	1.38^c^ ± 0.21	1.95^c^ ± 0.22	13.99^c^ ± 1.24	462.09^b^ ± 191.08	2554.60^a^ ± 117.06	40.913	0.000
- Zn	8.46^d^ ± 0.75	16.93^d^ ± 4.68	50.93^c^ ± 1.36	346.70^b^ ± 117.25	1899.47^a^ ± 220.30	45.571	0.000
- Mn	51.62^d^ ± 2.39	49.75^d^ ± 0.93	83.49^b^ ± 0.09	88.68^a^ ± 0.78	67.51^c^ ± 3.28	0.774	0.000

^a, b, c^ Means with different superscripts within a row are significantly different from each other at *p* < 0.05; SEM = standard error of the mean; *p*-value = probability value; Caf: calcite flour (Caf); Cag: calcite grit; CaP: calcite phosphate; eCa: enriched calcite; ecCa: enriched calcined calcite (ecCa).

As shown in Table 3, the iron (Fe) concentrations were consistently high across all formulas, except for ecCa, which showed a significantly reduced value. The ecCa formulas contained dramatically elevated levels of Cu and Zn, orders of magnitude higher than those in the non-enriched groups (Caf, Cag, and CaP), with the eCa formula serving as an intermediate. Finally, Mn was significantly higher in the eCa, followed by the CaP and ecCa formulas.

### 3.2. Particle size distribution and physical characteristics

The physical properties and particle size distributions of the calcite-based minerals are listed in Table 4. Cag exhibited the highest bulk density, whereas CaP exhibited the lowest. In contrast, the tapped bulk density was highest for Caf. Both Caf and Cag exhibited similar high specific density values, which were significantly greater than those of the phosphate-containing (CaP) and enriched (eCa and ecCa) formulas. In terms of handling and flowability, the angle of repose was significantly larger for Caf, CaP, eCa, and ecCa, indicating poorer flow characteristics. In contrast, Cag demonstrated a markedly lower angle, suggesting superior flow.

**Table 4.** Mean physical characteristics and particle size distribution of calcite-based minerals.

Parameters	Caf	Cag	CaP	eCa	ecCa	SEM	*p*-value
Physical properties							
- Bulk density (gm/ml)	1.29^c^ ± 0.01	1.45^a^ ± 0.01	1.22^d^ ± 0.01	1.30^c^ ± 0.02	1.34^b^ ± 0.01	0.006	0.000
- Tapped bulk density (gm/ml)	1.72^a^ ± 0.01	1.65^b^ ± 0.02	1.60^d^ ± 0.02	1.63^bc^ ± 0.02	1.63^cd^ ± 0.03	0.009	0.000
- Specific density (gm/ml)	2.16^a^ ± 0.16	2.17^a^ ± 0.18	1.35^b^ ± 0.05	1.39^b^ ± 0.07	1.46^b^ ± 0.10	0.045	0.000
- Angle of Repose (˚)	52.72^a^ ± 1.80	37.86^b^ ± 2.29	51.62^a^ ± 3.65	49.33^a^ ± 1.46	49.29^a^ ± 1.97	0.963	0.000
Particle size (%)							
- Coarse (≥ 1 mm)	4.47^b^ ± 0.58	3.83^c^ ± 0.49	1.60^e^ ± 0.07	5.22^a^ ± 0.08	2.78^d^ ± 0.51	0.171	0.000
- Medium (0.3–1 mm)	27.77^c^ ± 4.04	31.56^b^ ± 0.73	27.17^c^ ± 1.77	38.45^a^ ± 0.86	32.56^b^ ± 1.17	0.858	0.000
- Fine (< 0.3 mm)	67.76^a^ ± 4.46	64.62^b^ ± 1.10	70.93^a^ ± 1.55	56.33^c^ ± 0.50	64.65^b^ ± 1.39	0.926	0.000

^a, b, c^ Means with different superscripts within a row are significantly different from each other at *p* < 0.05; SEM = standard error of the mean; *p*-value = probability value; Caf: calcite flour (Caf); Cag: calcite grit; CaP: calcite phosphate; eCa: enriched calcite; ecCa: enriched calcined calcite (ecCa).

In terms of particle size and distribution, particle size analysis revealed that the eCa had the highest proportion of medium-sized particles and a significantly lower fine fraction than the other formulations, which were predominantly composed of fine particles.

### 3.3. Laying performances and physiological parameters

Table 5 summarizes the feeding trial results. No significant differences were observed in total and daily feed intake, average egg and eggshell weight, and egg index across all dietary treatments. Supplementation of the basal diet with local calcite-based minerals significantly (*p* < 0.05) improved laying quail performance, particularly egg production, feed efficiency, eggshell percentage, and eggshell thickness, compared with the control treatment. These improvements were associated with the higher dietary calcium supply provided by the supplemented diets. Among the supplemented groups, birds fed calcite grit (Cag, T2) achieved the highest egg production, followed by those fed calcite flour (Caf, T1) and enriched calcite (eCa, T4), resulting in greater egg mass and improved quail-day egg production.

**Table 5.** The mean feed intake, egg production, and eggshell quality, the weight of the liver and gizzard, and the profile of blood minerals and proteins (mean ± SD) of laying quails fed diets supplemented with calcite-based minerals.

Parameters	Experimental diets supplemented with minerals						SEM	*p*-value
	CMP	Caf	Cag	CaP	eCa	ecCa		
	T0	T1	T2	T3	T4	T5		
Feed intake (gm/bird)								
- Total feed intake^ns^	1586.77 ± 8.33	1618.97 ± 39.05	1620.56 ± 42.72	1618.86 ± 43.82	1599.79 ± 34.05	1608.25 ± 18.97	13.805	0.461
- Daily feed intake^ns^	28.34 ± 0.22	28.91 ± 0.71	28.94 ± 0.78	28.9 ± 0.74	28.57 ± 0.60	28.72 ± 0.34	0.248	0.461
Egg production								
- Egg number (egg/bird)	45.18^d^ ± 0.40	48.34^b^ ± 1.05	49.65^a^ ± 0.50	46.96^c^ ± 0.63	48.55^b^ ± 0.32	47.46^c^ ± 0.32	0.243	0.000
- Egg mass (gm/bird)	464.83^d^ ± 9.55	510.93^ab^ ± 14.04	519.67^a^ ± 5.98	493.31^c^ ± 1.73	509.31^b^ ± 4.03	494.21^c^ ± 8.58	3.404	0.000
- Quail-day egg production %)	80.68^e^ ± 0.64	87.12^b^ ± 2.41	88.66^a^ ± 0.78	82.81^d^ ± 0.30	86.70^b^ ± 0.57	84.75^c^ ± 0.52	0.456	0.000
Feed conversion ratio	3.42^a^ ± 0.06	3.17^cd^ ± 0.07	3.12^d^ ± 0.07	3.28^b^ ± 0.09	3.14^d^ ± 0.06	3.25^bc^ ± 0.04	0.028	0.000
Egg weight and egg index								
- Egg weight (gm/egg)^ns^	10.25 ± 0.17	10.39 ± 0.11	10.41 ± 0.10	10.37 ± 0.12	10.42 ± 0.07	10.29 ± 0.09	0.054	0.177
- Egg index^ns^	78.48 ± 0.55	78.16 ± 0.04	78.33 ± 0.24	79.00 ± 0.18	77.95 ± 0.88	78.04 ± 0.22	0.299	0.180
Egg shell quality								
- Eggshell weight (gm/egg)^ns^	0.786 ± 0.015	0.816 ± 0.009	0.802 ± 0.022	0.809 ± 0.006	0.806 ± 0.015	0.791 ± 0.011	0.007	0.125
- Percentage of eggshell (%)	7.17^b^ ± 0.11	7.28^ab^ ± 0.03	7.24^ab^ ± 0.09	7.28^ab^ ± 0.07	7.33^a^ ± 0.03	7.17^b^ ± 0.14	0.038	0.045
- Eggshell thickness(mm)	0.067^c^ ± 0.001	0.069^a^ ± 0.001	0.068^b^ ± 0.001	0.068^ab^ ± 0.001	0.068^b^ ± 0.000	0.067^c^ ± 0.001	0.000	0.029
Weight of liver and gizzard (gm/bird)								
- Liver	5.86^a^ ± 0.24	3.99^e^ ± 0.31	4.98^bc^ ± 0.08	5.14^b^ ± 0.11	4.31^d^ ± 0.33	4.79^c^ ± 0.23	0.097	0.000
- Gizzard^ns^	2.87 ± 0.14	2.86 ± 0.24	2.84 ± 0.33	3.24 ± 0.52	3.37 ± 0.29	3.08 ± 0.51	0.149	0.059
Blood mineral and protein (mg/dl)								
- Ca^ns^	6.57 ± 1.23	6.97 ± 1.10	6.40 ± 0.62	6.83 ± 1.18	8.33 ± 1.05	6.53 ± 1.34	0.452	0.054
- P	6.03^c^ ± 0.70	4.67^c^ ± 0.81	5.83^c^ ± 0.36	6.70^c^ ± 0.64	16.23^a^ ± 1.25	11.93^b^ ± 0.72	0.323	0.000
- Mg^ns^	3.07 ± 0.46	2.57 ± 0.33	3.23 ± 0.64	2.83 ± 0.34	3.17 ± 0.24	2.90 ± 0.14	0.161	0.066
Total protein (gm/dl)^ns^	3.27 ± 0.72	2.47 ± 0.29	3.90 ± 1.30	2.97 ± 0.27	3.43 ± 0.78	3.50 ± 0.83	0.319	0.065

^a, b, c ^Means with different superscripts within a row are significantly different from each other at *p* < 0.05; SEM = standard error of the mean; *p*-value = probability value; CMP: commercial mineral premix; Caf: calcite flour (Caf); Cag: calcite grit; CaP: calcite phosphate; eCa: enriched calcite; ecCa: enriched calcined calcite (ecCa).^ns^, non-significant.

Although differences in eggshell thickness and shell percentage among the supplemented treatments were relatively small, quails receiving Cag and Caf consistently showed numerically higher shell quality values than those fed the commercial mineral premix. The superior performance of Cag relative to the other supplemented treatments may indicate additional effects of particle size and physical characteristics on calcium utilization and eggshell formation. Consequently, these groups (T1, T2, and T4) exhibited significantly lower feed conversion ratios (FCR), indicating more efficient conversion of feed into egg mass than the control group.

Dietary treatment significantly affected liver weight (*p* < 0.001). Birds in the control group (T0) exhibited higher liver weights than those receiving local calcite-based mineral supplements. In contrast, gizzard weight remained unaffected by all treatments. Birds receiving diets supplemented with enriched calcite (eCa, T4) and enriched calcined calcite (ecCa, T5) exhibited the highest plasma P concentrations (*p* < 0.001), consistent with the elevated P content of these formulations (Table 3). No significant differences were observed in the blood levels of Ca, Mg, or total protein.

### 3.4. Economic performance

The partial budget analysis presented in Table 6 indicates variation in economic efficiency among the dietary treatments, with some local calcite-based mineral supplements yielding higher net returns than the commercial premix. The total gross return was highest for the groups fed calcite grit (Cag, T2), followed by those fed enriched calcite (eCa) and calcite flour (Caf, T1), primarily due to their superior egg sales revenue. Concurrently, the Caf and Cag formulations had the lowest total variable costs, which were inversely related to the level of enrichment, with the non-enriched Caf (T1) and Cag (T2) being the most economical and the commercial premix (T0) being the most expensive. This combination of higher revenue and lower cost resulted in substantially higher net returns for the Cag (T2) and Caf (T1) groups.

**Table 6.** Partial budget and marginal analysis of an 8-week feeding trial with laying quails, arranged by ascending variable cost (IDR*/bird).

Items	Experimental diets supplemented with minerals					
	Caf	Cag	CaP	eCa	ecCa	CMP
	T1	T2	T3	T4	T5	T0
Gross return (IDR/bird)						
Carcass	8,000.0	8,000.0	8,000.0	8,000.0	8,000.0	8,000.0
Manure	634.2	648.4	590.0	630.7	653.6	626.4
Egg	14,503.0	14,895.5	14,089.4	14,565.2	14,238.6	13,553.5
Total gross return	23,137.3	23,543.9	22,679.4	23,195.8	22,892.2	22,181.0
Variable cost (IDR/bird)						
Feed cost	11608.0	11698.5	11773.5	11921.1	12079.5	12257.8
Capital cost	232.2	234.0	235.5	238.4	241.6	245.2
Total variable cost	11,840.2	11,932.4	12,009.0	12,159.6	12,321.1	12,502.9
Net return	11,297.1	11,611.5	10,670.4	11,036.3	10,571.1	9,678.1
Marginal cost		92.3	76.6	150.5	161.6	181.8
Marginal net return		314.4	(-)	365.9	(-)	(-)
Marginal rate of net return		3.4	(-)	2.4	(-)	(-)

* IDR: Indonesian Rupiah; CMP: commercial mineral premix; Caf: calcite flour (Caf); Cag: calcite grit; CaP: calcite phosphate; eCa: enriched calcite; ecCa: enriched calcined calcite (ecCa).

**Figure 1.**
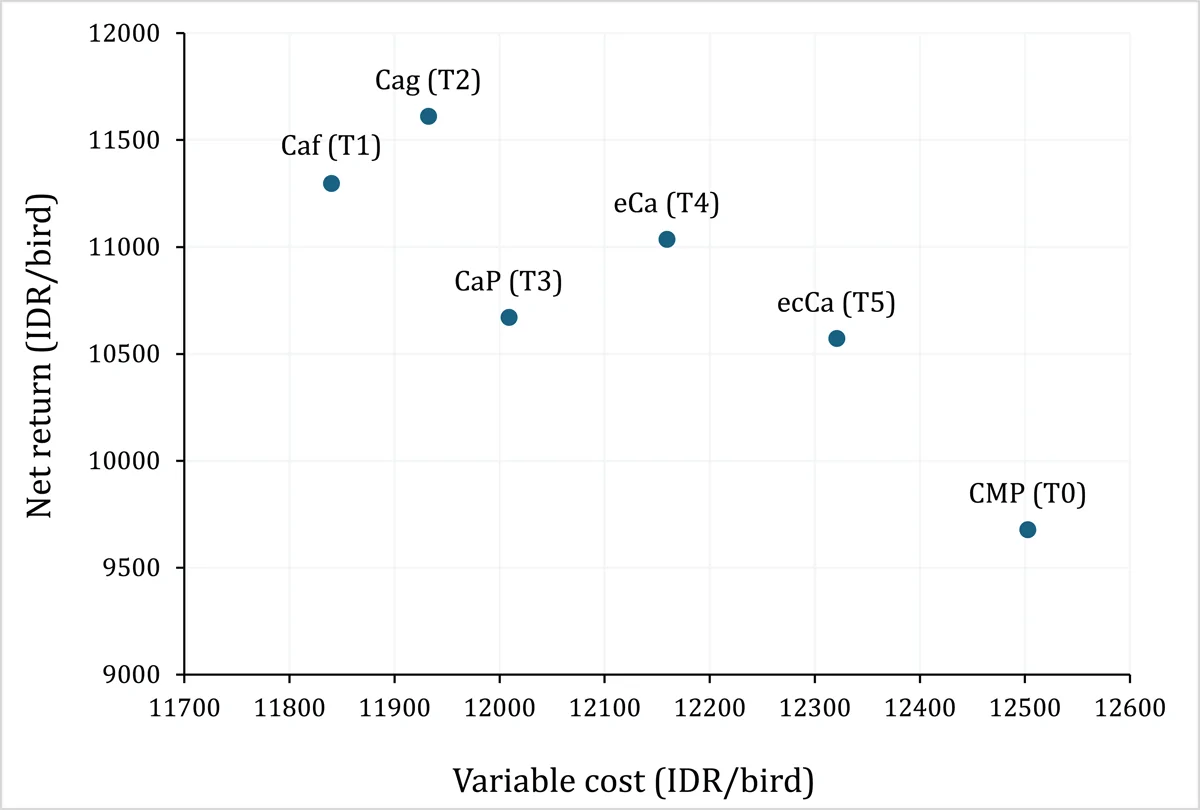
Dominance analysis of variable cost and net return [IDR: Indonesian rupiah; CMP: commercial mineral premix; Caf: calcite flour (Caf); Cag: calcite grit; CaP: calcite phosphate; eCa: enriched calcite; ecCa: enriched calcined calcite (ecCa)].

Based on marginal analysis, transitioning from the baseline Caf (T1) diet to the Cag (T2) diet incurred a small additional cost, resulting in the highest marginal rate of return (3.4), i.e., 3.4 IDR net return per each IDR additional cost incurred when the Cag was selected instead of Caf (Table 6). Therefore, if capital is available, Cag (T2) is a more cost-effective choice than Caf (T1). As shown in Figure 1, the dominance analysis confirms that both Caf and Cag are economically dominant, with Cag being the most cost-effective mineral supplement for maximizing profit in laying quail feeding.

## 4. Discussion

Calcium, the predominant mineral in the local calcite formulations, was highest in the raw calcite flour (Caf) and calcite grit (Cag) treatments. Together with their favorable physical properties, these characteristics support the potential use of local calcites as practical calcium sources for laying quails [21]. However, interpretation of the productive responses requires caution because the experimental diets were not formulated to be isocalcium or isomineral. The control diet contained substantially lower calcium (2.0%) than the calcite-supplemented diets (2.7–3.0%). Therefore, part of the improved productive performance observed in T1-T5 may reflect the correction of inadequate calcium supply rather than differences in mineral-source quality alone.

Despite the more favorable Ca/P ratio in the control diet, quails receiving the calcite-based supplements exhibited higher egg production, improved feed conversion, and better eggshell characteristics. These responses emphasize the importance of adequate calcium supply for eggshell formation and productive performance in laying birds. Laying quails require relatively high dietary calcium because approximately 2 gm of calcium is deposited daily into eggshells during active production, and inadequate calcium intake rapidly impairs shell mineralization and egg output [1]. Previous studies have demonstrated that insufficient dietary calcium exerts a greater negative effect on laying performance than moderate variation in dietary phosphorus concentration, provided that phosphorus remains within the physiological requirement range [22].

Differences in calcium particle characteristics may also have contributed to the observed responses. Fine-particle calcium sources dissolve rapidly and are readily available for intestinal absorption. In contrast, coarse particles remain longer in the gizzard and provide a slower and more sustained release of calcium during nocturnal eggshell calcification when feed intake is limited [23, 24]. This mechanism is particularly important in laying poultry because shell mineralization occurs mainly during the dark period [25]. In the present study, quails fed calcite grit (Cag) showed numerically superior productive performance and shell quality compared with the enriched and calcined formulations. Nevertheless, the independent contribution of particle size could not be isolated because dietary calcium concentration, Ca/P ratio, and mineral enrichment varied simultaneously across treatments. Therefore, the present findings support only a possible contribution of calcium physical characteristics to mineral utilization and eggshell deposition rather than definitive causal effects.

The favorable performance of calcite grit was also associated with its physical properties. Calcite grit exhibited the highest bulk density, reflecting its coarse particulate structure and reduced inter-particle voids. Coarse particles generally improve packing efficiency [26] and retention within the gastrointestinal tract, thereby prolonging mineral availability in the gizzard [24]. In contrast, calcite phosphate (CaP) exhibited lower bulk density because of the inclusion of bone meal and other lower-density components. Similarly, the higher specific density observed in Cag and Caf reflected the predominance of dense mineral-rich calcite particles. In contrast, lower values in CaP, eCa, and ecCa were associated with the incorporation of phosphates, salts, and trace mineral additives.

Calcite grit also exhibited the lowest angle of repose, indicating superior flowability and improved handling and mixing characteristics [27]. Adequate flowability is important for achieving more uniform mineral distribution in poultry diets and reducing segregation during feed preparation and storage [28]. In addition, the lower tapped bulk density of Cag compared with Caf was consistent with the tighter compaction behavior of fine powders, in which smaller particles reduce interstitial air space during compression [28]. Collectively, these physical characteristics may have contributed to differences in mineral utilization efficiency among the tested formulations.

Birds fed non-enriched local calcites, particularly calcite flour (Caf), exhibited significantly lower liver weights than those receiving the commercial mineral premix. However, interpretation of this response should be cautious, as hepatic enzymes, oxidative stress indicators, renal biomarkers, and histopathological evaluations were not measured in the present study. Therefore, the lower liver weight observed in the non-enriched treatments cannot be directly interpreted as evidence of reduced metabolic burden or improved hepatic health. Additional toxicological and histological evaluations are required before functional conclusions regarding liver status can be established.

Differences in mineral particle characteristics among treatments may also have influenced mineral solubility, gastrointestinal retention, and digestive kinetics [29, 30]. Coarse mineral particles generally remain longer in the gizzard and release calcium more gradually than fine particles, thereby influencing calcium availability during eggshell mineralization [24]. Nevertheless, these physiological mechanisms were not directly evaluated in the present study. Gizzard weight did not differ significantly among treatments, indicating that inclusion of coarse mineral particles influenced mineral utilization without substantially altering organ size [24].

Enrichment and calcination increased phosphorus, sodium, zinc, and copper concentrations in the CaP, eCa, and ecCa formulations to levels that met or exceeded avian mineral requirements [31]. The Cu and Zn concentrations reported in Table 3 refer to the mineral supplement formulations rather than the final complete diets, as the supplements were included at only 5% of the total ration. Consequently, the actual dietary concentrations of these trace minerals were substantially lower than the values reported for the mineral mixtures. Nevertheless, the enriched formulations still supplied markedly higher concentrations of Cu and Zn than the non-enriched treatments, and excessive dietary trace minerals can interfere with mineral metabolism, impair iron absorption, increase oxidative stress, and alter hepatic function in poultry when supplied beyond physiological requirements [32, 33].

Calcination may also have influenced the physicochemical characteristics of the local calcites. Thermal treatment at high temperatures alters mineral crystallinity, removes organic matter and bound water, and increases mineral brittleness and porosity, thereby affecting particle structure and solubility [13]. Calcination of calcium carbonate converts part of the material into calcium oxide, which is generally more reactive and rapidly solubilized under acidic gastrointestinal conditions [34]. Faster dissolution may improve short-term calcium availability but can also reduce gizzard retention time compared with coarse and less soluble particles. In laying poultry, prolonged retention of coarse calcium particles in the gizzard is considered beneficial because it provides sustained calcium release during nocturnal eggshell mineralization [24].

These physicochemical changes may partly explain why the calcined and enriched formulations did not outperform calcite grit (Cag) in egg production or eggshell quality despite their higher mineral concentrations. The superior performance of Cag suggests that physical characteristics, such as particle size, hardness, density, and retention behavior, may exert a greater influence on calcium utilization efficiency than additional mineral fortification alone. Similar observations have been reported in laying hens, where coarse and slowly soluble calcium sources improved eggshell quality more consistently than highly soluble fine-particle, highly soluble sources [18, 23]. However, mineral crystallinity, *in vitro* solubility, gastrointestinal retention time, and true calcium bioavailability were not directly measured in the present study. Therefore, the proposed effects of calcination on mineral utilization remain interpretative and should be confirmed through future studies evaluating mineral dissolution kinetics, gastrointestinal retention behavior, and long-term physiological responses in laying poultry.

The significantly higher serum phosphorus concentrations observed in birds fed eCa and ecCa reflected the high phosphorus availability of dicalcium phosphate and bone meal incorporated into these formulations [35]. In contrast, blood Ca, Mg, and total protein concentrations did not differ significantly among treatments, indicating that no measurable alterations were detected in these variables during the experimental period. However, these blood parameters alone are insufficient to evaluate mineral safety or chronic metabolic stress comprehensively. The absence of hepatic enzyme measurements, renal injury biomarkers, tissue mineral accumulation analysis, oxidative stress indicators, and organ histopathology, therefore, represents an important limitation of the present study.

The improved feed conversion ratio observed in eCa may be associated with the physiological roles of Zn and Cu in enzymatic activity, mineral metabolism, and eggshell formation. However, excessive concentrations of trace minerals can also produce antagonistic interactions among Zn, Cu, and Fe, thereby reducing mineral utilization efficiency and potentially impairing productive performance. Such interactions may partly explain why the enriched and calcined formulations did not surpass calcite grit (Cag) in egg production or economic return, despite their higher supplemental mineral concentrations. Similar antagonistic relationships among trace minerals have previously been reported in poultry nutrition studies [36, 37].

The decline in calcium concentration from Caf and Cag to CaP, eCa, and ecCa reflected increasing inclusion of non-calcite ingredients, including phosphate sources and trace mineral additives. Nevertheless, dietary calcium concentrations in all supplemented treatments remained within the range generally considered adequate for eggshell formation in laying quails [6]. Similarly, the relatively low phosphorus concentrations in Caf and Cag did not impair productive performance, indicating that phosphorus supplied by the basal diet was adequate under the conditions of the present experiment. Therefore, the present findings suggest that adequate calcium supply and the physical characteristics of local calcites, particularly particle size and hardness, exerted greater influence on productive performance than additional trace mineral fortification.

The partial budget analysis indicated that local calcite-based mineral supplements improved economic efficiency under the experimental conditions of the present study. Birds fed calcite grit (Cag) achieved the highest net return, followed by those fed calcite flour (Caf), primarily because of improved egg production, higher egg revenue, and lower variable feed costs. These findings are consistent with previous studies demonstrating that locally available calcium sources, including limestone, oyster shell, and eggshell-derived minerals, can reduce feed costs while maintaining productive performance in laying poultry [38]. The relatively low production costs of Caf and Cag were associated with the availability of inexpensive raw materials and minimal processing requirements, thereby increasing their practical value for smallholder quail production systems [39, 40]. Improved egg production in birds receiving Caf and Cag also contributed to higher gross returns, supporting previous observations that productive efficiency is a major determinant of profitability in laying operations [41]. In addition, the use of locally available calcite resources may reduce dependence on imported commercial mineral supplements and contribute to more regionally sustainable utilization of feed resources.

The present study demonstrated that the mineral formulas commonly used by small-scale quail farmers may not adequately meet the mineral requirements of laying quails, particularly calcium requirements associated with sustained egg production and eggshell formation, thereby contributing to suboptimal productive performance and economic returns. Locally sourced calcite-based mineral supplements, especially calcite grit, improved laying performance and economic efficiency without detectable short-term adverse effects on the measured physiological variables, highlighting their potential as practical and affordable alternative mineral sources for smallholder quail production systems.

Nevertheless, the present findings should be interpreted cautiously because natural calcite resources can vary considerably in mineral composition, particle characteristics, impurity levels, and heavy-metal content depending on geological origin and processing methods, which may influence nutrient consistency, bioavailability, and long-term safety under commercial conditions. Furthermore, broader evaluations of long-term toxicity, tissue mineral accumulation, hepatic and renal function, oxidative stress responses, organ histopathology, and economic sensitivity under fluctuating market conditions were beyond the scope of the present experiment. Therefore, further large-scale and on-farm investigations are required to validate standardization of processing, toxicological safety, mineral consistency, and commercial feasibility before the widespread application of local calcite-based mineral supplements in laying poultry production can be fully recommended.

## 5. Conclusions

This study suggests that calcite grit (Cag) was the most economically favorable mineral supplement for laying quails, delivering the highest net returns while maintaining production performance. Productive performance was more strongly influenced by physical characteristics, particularly coarse particle size and density, than by calcination or mineral enrichment. These findings offer a practical, sustainable, and cost-effective strategy for improving local farmers’ incomes while utilizing indigenous mineral resources. Future studies should evaluate the toxicological safety of the most promising formulas (Cag and Caf) and validate their practical applicability through on-farm trials using semi-self-mixed diets, while exploring their potential commercialization as sustainable local mineral supplements for quail production.

## Data Availability

The data presented in this study are available from the corresponding author upon reasonable request.

## List of abbreviations

Caf, calcite flour; Cag, calcite grit; CaP, calcite phosphate; CMP, commercial mineral premix; eCa, enriched calcites; ecCa, enriched calcined calcites; FCR, feed conversion ratio; IDR, Indonesian Rupiah; SD, standard deviation; SEM, standard error of the mean; gm, grams; h, hours; min, minutes; ml, milliliters; cm, centimeter; gm/ml, grams per milliliter.
